# The Paradox of Hyperkalaemia: When Treatment Isn't the Answer

**DOI:** 10.7759/cureus.68727

**Published:** 2024-09-05

**Authors:** Abdul Bhat, Amy Turnbull, Abir Aijaz, Amit Badshah

**Affiliations:** 1 Internal Medicine, University Hospitals Bristol and Weston, Weston-super-Mare, GBR; 2 Acute and General Internal Medicine, University Hospitals Bristol and Weston, Weston-super-Mare, GBR

**Keywords:** deep vein thrombosis (dvt), leucocytosis, plasma potassium, pseudohyperkalemia, serum potassium level, thrombocytosis

## Abstract

Hyperkalaemia is a relatively common medical emergency that necessitates prompt and urgent intervention. There is an ongoing debate over the precise threshold for treating hyperkalaemia due to variability in clinical scenarios. This case report highlights the need to differentiate true hyperkalaemia from pseudohyperkalaemia by analysing serum and plasma potassium levels, thus avoiding unnecessary treatment and the risk of iatrogenic hypokalemia. This case report discusses an 89-year-old male who presented with recurrent falls and fluctuating serum potassium levels but showed no symptoms of hyperkalaemia and had no relevant drug history. Further investigation revealed an underlying myeloproliferative neoplasm with thrombocytosis, leading to the diagnosis of pseudohyperkalaemia, reflected by a significant discrepancy between serum and plasma potassium levels, showcasing the importance of considering pseudohyperkalaemia in patients with haematological malignancies and thrombocytosis.

## Introduction

Hyperkalaemia is a potentially life-threatening emergency, hence it is vital to recognize it early for prompt initiation of treatment. Hyperkalaemia is defined as a serum potassium level greater than 5.5 mmol/litre [1]. Serum levels greater than 6.5 mmol/litre or lower, if associated with ECG changes, should trigger the initiation of urgent treatment [2].

Relatively small changes in extracellular potassium can cause significant abnormalities predominantly associated with neuromuscular and cardiac dysfunction. Despite national guidelines, controversy remains around the exact levels that should initiate treatment due to variations in optimal levels in different conditions. Furthermore, the pitfalls of both recognising and managing hyperkalaemia may result in additional complications [2,3].

An artificial rise in serum potassium, known as pseudohyperkalaemia is important to recognize because treatment may result in severe iatrogenic hypokalemia, with a risk of life-threatening arrhythmias and increased mortality [4].

We present a case of an 89-year-old male who was thought to have hyperkalaemia, which was treated as per protocol. Subsequently, he developed hypokalemia reflecting the importance of detecting pseudohyperkalaemia, which demonstrates the importance of considering the clinical context of repeated high serum potassium, particularly when refractory to medical treatment.

## Case presentation

An 89-year-old male was referred to the emergency department by his general practitioner who noted a serum potassium level of 7.2 mmol/L (Table 1) during routine blood tests. The patient had experienced a six-month history of recurrent falls, reduced appetite, and general functional decline with decreased mobility due to generalized body weakness.

He also reported intermittent dysuria and urinary frequency over the past five weeks. His medical history was significant for polymyalgia rheumatica (on long-term steroids - Prednisolone 10 milligram once a day), osteoarthritis, amaurosis fugax, transient ischemic attack, iron deficiency anaemia, benign prostatic hyperplasia, cervical spondylosis, and a history of gastric cancer treated with gastrectomy six years ago when he was deemed cancer free. His baseline blood was as under with a clear disparity between plasma and serum potassium noted.

On examination, his blood pressure was 136/86 mmHg, pulse was 80 per minute and regular, respiratory rate was 14 per minute and saturation was 97% on room air. He appeared frail, cachectic, and dehydrated, with pitting oedema up to the mid-shin and mild swelling, erythema and tenderness in the right calf. Initial tests showed a discrepancy between plasma potassium (4.0 mmol/L) and serum potassium (7.2 mmol/L) (Table 1). The patient also had leucocytosis, thrombocytosis, and a slight decrease in the glomerular filtration rate (Table 1). Despite the absence of ECG changes typically associated with hyperkalaemia, the on-call team administered standard hyperkalaemia treatment, resulting in hypokalaemia.

**Table 1:** Lab values mmol/l – millimol per litre; mg/L – milligram per litre; WBC – white blood cells; EGFR – estimated glomerular filtration rate; CRP – C-reactive protein

PARAMETERS	PATIENT RESULTS	REFERENCE RANGE
Serum potassium	7.2 - Day 1, 6.5 - Day 2, 6.7 - Day 3	3.5-5.5 mmol/l
Plasma potassium	4.0 - Day 2, 3.9 - Day 3, 3.1 - Day 4	3.5-5.5 mmol/l
Platelets	1944 - Day 2, 867 - Day 4	10^9^/L
WBC	14.1 - Day 1, 19.2 -Day 2, 11.3 - Day 4	4-11 10^9^/L
EGFR	76	>90 ml/min
CRP	<5	<5 mg/L
Sodium	136	135-145 mmol/l

**Figure 1:**
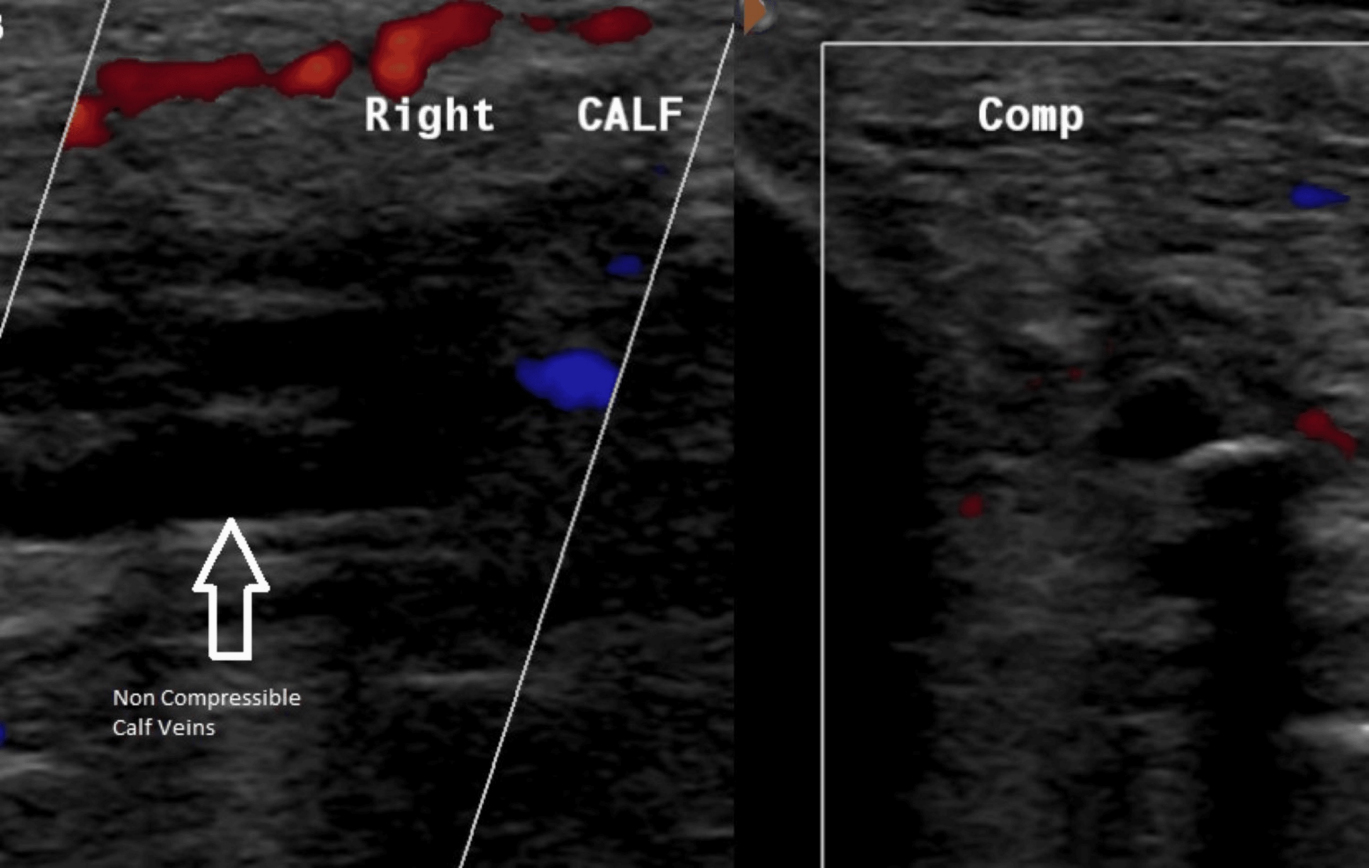
Doppler of right calf showing non-compressible veins – thrombus (white arrow) Comp – compression Doppler image

He was also treated for a urinary tract infection with co-amoxiclav for five days and given low molecular weight heparin for suspected deep vein thrombosis, later confirmed by a duplex scan (Figure 1).

**Figure 2:**
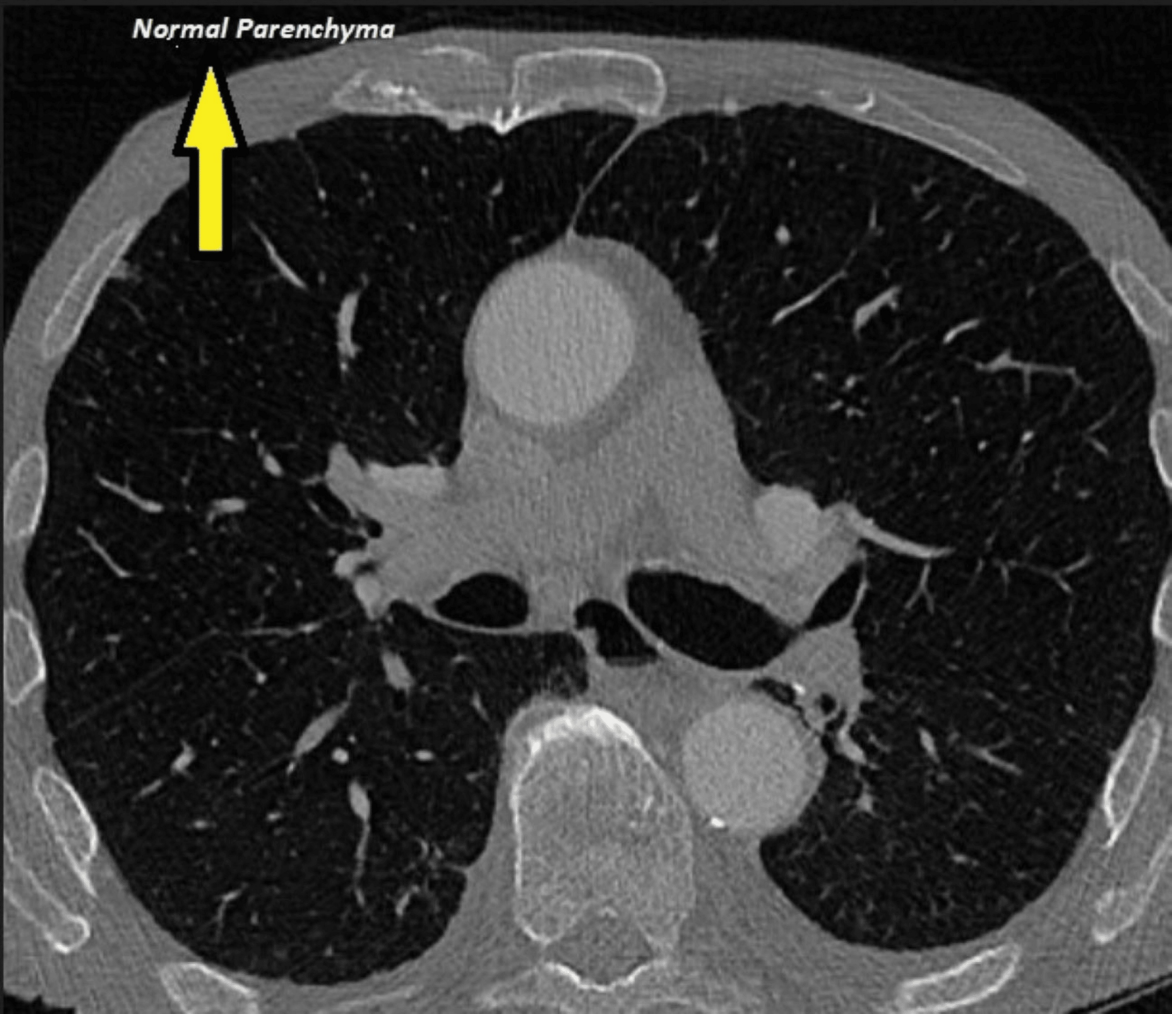
CT tap suggestive of normal lung parenchyma and thorax (black arrow) CT tap – computed tomography of thorax, abdomen and pelvis

Subsequent tests showed a persistently elevated serum potassium of 6.7 mmol/L, prompting further hyperkalaemia treatment. Computed tomography of the thorax, abdomen, and pelvis (Figure 2) was also done to rule out any indwelling cancer and was reported as normal.

Given the thrombocytosis and leucocytosis, additional tests, including JAK2 and BCR-ABL, were performed as suspicion of a myeloproliferative disease was high.

The patient was eventually diagnosed with a JAK2-positive myeloproliferative neoplasm with essential thrombocythemia and he was treated with hydroxycarbamide with a plan to give radioactive phosphorus as per haematology review.

The patient did not need any treatment for pseudohyperkalaemia after it was established, which prevented him from further iatrogenic hypokalemic episodes.

 This case highlights the importance of considering pseudohyperkalaemia in patients with thrombocytosis linked to myeloproliferative disorders and always keeping in mind the rare manifestations of relatively common diseases.

## Discussion

Hyperkalaemia is a potentially life-threatening and clinically one of the most important electrolyte disturbances encountered in the hospital setting. Its incidence, although likely underestimated, has been reported as 0.4 cases per 100-person years in the general population [5]. Hyperkalaemia may result from many underlying conditions and drugs. Chronic kidney disease is one of the main risk factors for developing hyperkalaemia in the general population. The other conditions include heart failure, cerebrovascular disease and diabetes mellitus due to type 4 renal tubular acidosis. Drugs and medications frequently causing hyperkalaemia include potassium-sparing diuretics, angiotensin-converting enzyme (ACE) inhibitors, non-steroidal anti-inflammatory drugs (NSAIDs) and angiotensin receptor blockers (ARBs).

Despite mechanisms for potassium homeostasis, hyperkalaemia may result from extracellular potassium shift, increased intake and reduced renal excretion [6]. Potassium is the major intracellular cation and is concentrated by Na+ - K+ ATPase in the plasma membrane. The Na+ - K+ ATPase pumps, three Na+ ions out of the cell in exchange for two K+ ions by hydrolysing ATP. The resting membrane potential of nerve and muscle cells depends on the difference between extra- and intracellular potassium ion concentrations, which, in turn, determines the excitability. Cells have a high selective permeability to potassium ions and, therefore, generate a negative resting membrane potential. This stabilises atrial and ventricular myocytes during diastole preventing spontaneous action potentials, which would otherwise result in premature extra-systoles [7].

Cardiac arrhythmias result from the direct effect of potassium, as well as the balance of Na+, Ca2+ and K+ [7]. As determined by the Nernst equation, in hyperkalaemia, the K+ equilibrium potential (Ek) becomes less negative. Increased K+ channel conductance results in a shortened action potential duration, which eventually prolongs the effective refractory period and enhances the repolarisation reserve(7). Furthermore, conduction through the AV node may be impaired and the pace-making function of His-Purkinje tissue is suppressed because it has a higher resting K+ conductance, predisposing to asystole. Early repolarisation of the action potential secondary to increased repolarisation reserve predisposes to further arrhythmias [7]. Consequently, relatively small changes in extracellular potassium can adversely affect neuromuscular and cardiac function [8]. There is wide variability of ECG changes associated with hyperkalaemia, including shortened QT interval, peaked T waves, QRS prolongation, shortened PR interval, and a reduction in P wave amplitude. In severe cases, the classic ‘sine-wave’ ventricular rhythm may result due to loss of sinoatrial conduction. ECG may also demonstrate heart block, asystole, and ventricular tachycardia or fibrillation [7]. A retrospective study demonstrated poor sensitivity and specificity of ECG changes, but the probability of changes increased with higher serum potassium levels [9]. This highlights the importance of clinical context and serial measurements of serum potassium.

There is significant variation in the clinical presentation of hyperkalaemia [10]. Patients may be asymptomatic or present with fatigue, dyspnoea, nausea, vomiting, abdominal pain and chest pain [10]. The lack of correlation between clinical presentation, ECG changes and serum potassium level often leads to challenges in diagnosis.

Pseudohyperkalaemia, also called spurious/factitious/artifactual hyperkalaemia, is defined as elevated potassium level in the local blood vessel or in vitro, caused by potassium release during or post venipuncture, with no physiological consequences. Pseudohyperkalaemia refers to those conditions in which the elevation in the measured serum potassium concentration is usually due to potassium movement out of the cells during or after the blood specimen has been drawn [6,7,11].

This phenomenon was first described by Hartmann and Mellinoff in 1955, where serum potassium levels were markedly greater than plasma levels with no associated symptoms. This was attributed to the release of potassium from platelets during aggregation and degranulation [12].

The serum and plasma potassium concentrations differ by greater than 0.4 mmol/l samples taken at the same time, at room temperature and tested within one hour of collection of samples [13]. It is important to recognise because this artifactual increase may result in a false interpretation of an elevated serum potassium level, and consequently treatment to lower the potassium level may cause severe hypokalaemia. The prevalence of pseudohyperkalaemia is reported as up to 40%, particularly in the context of leukocytosis [14].

The most common cause of pseudohyperkalaemia is in vitro haemolysis, which may be precipitated by thrombocytosis, leucocytosis or erythrocytosis due to release from cellular elements. In addition, venipuncture methods, such as tourniquet use, contraction of the forearm and clenching of the fist, may increase potassium excretion from muscle. Potassium ethylenediaminetetraacetic acid (EDTA) may contaminate samples and sample handling methods resulting in mechanical trauma to erythrocytes may all cause a spurious result. Extracellular potassium shift may result from respiratory alkalosis secondary to acute anxiety and hyperventilation during venipuncture and potassium may be redistributed in cachectic patients due to altered T tube architecture in skeletal muscle. Some genetic subtypes are related to erythrocyte permeability, which is temperature dependent [6-9,15].

The phenomenon of pseudohyperkalaemia has been reported in cases of chronic lymphocytic leukaemia and severe leucocytosis. Leukaemia lymphocytes are fragile and release potassium during centrifugation, tube transport in the hospital, and when shaken. This explains why the potassium level may be higher in plasma than in serum. The interaction with lithium heparin in plasma blood samples may also result in higher cell membrane permeability, and high leukocyte counts may also contribute due to depletion of metabolites resulting in impaired Na-K ATPase activity and the release of K+ [15]. Therefore, diagnosing pseudohyperkalaemia is a particular problem in this cohort of patients.

Thrombocytosis is associated with hyperkalaemia since platelet activation is related to potassium release during clot formation. In previous cases, the greatest discrepancy in serum and plasma potassium levels has been in patients with thrombocytosis [16]. An artificial increase of 0.45 mmol/L has been reported in chronic myelocytic leukaemia and 0.87 mmol/L in essential thrombocytosis, by Sevastos et al. [16]. In our case, the patient had significant thrombocytosis at presentation with no obvious cause. This was later investigated and explained by a myeloproliferative neoplasm, which explains the pseudohyperkalaemia that subsequently resulted in iatrogenic hypokalaemia.

Familial pseudohyperkalaemia sometimes called leaky cell syndrome describes a particular and rare inherited presentation of pseudohyperkalaemia characterised by erythrocyte membrane structure defects. The gene responsible is ABCB6 and the mode of inheritance is autosomal dominant. Affected individuals are usually asymptomatic. This may also explain the laboratory findings in this case. In addition, it was important to consider tumour lysis syndrome, which may also present in patients with leukaemia with hyperkalaemia as well as hyperuricaemia, hyperphosphatemia and hypocalcaemia [13].

## Conclusions

This case reflects the importance of considering clinical context when evaluating serum potassium levels, especially in patients without ECG changes. Patients who present with thrombocytosis and leukocytosis, pseudohyperkalaemia can mimic true hyperkalaemia, potentially leading to inappropriate untimely treatment and resulting in severe iatrogenic hypokalaemia. Therefore, when hyperkalaemia is noted without corresponding ECG abnormalities, clinicians should maintain a high index of suspicion for pseudohyperkalaemia and consider serial measurements using heparinized or whole blood samples to check plasma potassium. Further research is needed to establish guidelines for identifying pseudohyperkalaemia in high-risk patients, such as those with myeloproliferative disorders, as it can be potentially critical for the health of the patient.
